# Global health classroom: mixed methods evaluation of an interinstitutional model for reciprocal global health learning among Samoan and New Zealand medical students

**DOI:** 10.1186/s12992-021-00755-8

**Published:** 2021-09-03

**Authors:** Roshit K. Bothara, Malama Tafuna’i, Tim J. Wilkinson, Jen Desrosiers, Susan Jack, Philip K. Pattemore, Tony Walls, Faafetai Sopoaga, David R. Murdoch, Andrew P. Miller

**Affiliations:** 1 Department of Pathology and Biomedical Science, University of Otago, PO Box 4345, Christchurch, 8140 New Zealand; 2 School of Medicine, National University of Samoa, Apia, Samoa; 3 Department of Medicine, University of Otago, Christchurch, New Zealand; 4 Department of Population Health, University of Otago, Christchurch, New Zealand; 5 Department of Preventive and Social Medicine, University of Otago, Dunedin, New Zealand; 6 Department of Paediatrics, University of Otago, Christchurch, New Zealand; 7 Centre for Pacific Health, Va’a o Tautai, Division of Health Sciences, University of Otago, Dunedin, New Zealand

**Keywords:** Global health education, Digital technology, Videoconferencing, Partnership, Collaboration

## Abstract

**Background:**

Global health education partnerships should be collaborative and reciprocal to ensure mutual benefit. Utilisation of digital technologies can overcome geographic boundaries and facilitate collaborative global health learning.

Global Health Classroom (GHCR) is a collaborative global health learning model involving medical students from different countries learning about each other’s health systems, cultures, and determinants of health via videoconference. Principles of reciprocity and interinstitutional partnership informed the development of the GHCR. This study explores learning outcomes and experiences in the GHCR between students from New Zealand and Samoa.

**Methods:**

This study used a mixed methods approach employing post-GHCR questionnaires and semi-structured face-to-face interviews to explore self-reported learning and experiences among medical students in the GHCR. The GHCR collaboration studied was between the medical schools at the University of Otago, New Zealand and the National University of Samoa, Samoa.

**Results:**

Questionnaire response rate was 85% (74/87). Nineteen interviews were conducted among New Zealand and Samoan students. Students reported acquiring the intended learning outcomes relating to patient care, health systems, culture, and determinants of health with regards to their partner country. Interview data was indicative of attitudinal changes in relation to cultural humility and curiosity. Some reported a vision for progress regarding their own health system. Students in the GHCR reported that learning with their international peers in the virtual classroom made learning about global health more real and tangible. The benefits to students from both countries indicated reciprocity.

**Conclusions:**

This study demonstrates GHCR to be a promising model for collaborative and reciprocal global health learning using a student-led format and employing digital technology to create a virtual classroom. The self-reported learning outcomes align favourably with those recommended in the literature. In view of our positive findings, we present GHCR as an adaptable model for equitable, collaborative global health learning between students in internationally partnered institutions.

**Supplementary Information:**

The online version contains supplementary material available at 10.1186/s12992-021-00755-8.

## Introduction

Global health learning is increasingly recognised as an essential component of undergraduate medical curricula [1, 2]. Doctors of the future must have the relevant knowledge, attitudes, and skills to practise in an increasingly interconnected world where health inequities persist [1, 3]. However, there is a lack of consensus on the required key competencies and most effective models for global health learning [4–6]. Major work has been done by organisations and committees, such as the Consortium of Universities for Global Health and Bellagio Global Health Education Initiative to standardise global health curricula and delivery methods [5, 7].

Active and transformative global health learning models, that focus on competencies related to determinants of health and cultural humility and curiosity, have been recommended [7–9]. The most common global health education approaches are didactic, such as lectures and tutorials, often supplemented by opportunities for international field electives mainly for students from high income countries [4, 10]. Educators have called for an integrated and transformative approach to global health learning, where global health concepts are integrated into curricula, rather than heavy dependence on international field electives [8]. Electives can be very effective for medical students to learn experientially about global health, particularly to gain knowledge of diseases less common in their home country, to learn about other cultures and health systems, and for self-development [1, 2, 11, 12]. Typically, such electives involve medical students from high-income countries travelling to middle- or low-income countries where the benefits to the host institutions are less certain [13]. The lack of reciprocity has raised concerns about short-term international field electives, particularly in the absence of equitable partnerships, as these may perpetuate attitudes of cultural and professional superiority. Other concerns regarding electives include commercialisation, inadequate pre- and post-elective briefings, and personal safety risks [12–17]. As well, the objectives of international field electives for medical students from high-income countries do not consistently align with the priorities and needs of hosting middle- and low-income countries [14]. Importantly, opportunities for students in low-income countries to undertake similar international electives are limited, particularly due to financial constraints [13].

Recognising this lack of reciprocity, the objectives, effectiveness, and ethics of experiential global health learning through international electives have been called into question [14, 18]. In response, ethical and practical guidelines have been proposed with recommendations that international electives are best done within formal interinstitutional partnerships in order to better align learning expectations, safety and mutual benefits for the hosts and visiting students [12, 18, 19]. However, even within formal partnerships, students from low-income countries typically are unable to travel to high income countries for similar learning experiences. Global health is rooted in equity and key values are reciprocity and collaboration [14, 20]. Therefore, pedagogies for global health education within international partnerships must be developed collaboratively to fit the needs, interests, and limitations of all participating institutions and students, regardless of their locations [5, 14].

In their landmark 2010 report, Frenk and colleagues encouraged the redesign of health professional education, recommending the use of digital technologies to foster reciprocal and collaborative partnerships, harness global knowledge and enable transformative learning among students [3]. Digital technology can transcend geographic boundaries to connect students internationally for mutual learning about their partner country’s health system, cultures, and determinants of health. Despite the almost global availability and ever increasing capability of digital technologies, there are limited reports of health professional training institutions linking in real time for bilateral, collaborative transnational global health education [21, 22]. Currently, the small number of published models show much potential for such collaborations but are either pilot studies or have not been integrated into medical curricula [21, 22].

In this report we describe a novel global health learning model, Global Health Classroom (GHCR), which was developed between Otago Medical School at the University of Otago, New Zealand (OMS) and the School of Medicine at the National University of Samoa, Samoa (NUS). Given there are many definitions of global health, we considered the definition by Koplan et al. most appropriate to our study because of its emphasis on equity. Development of GHCR, both as a pedagogy and as an interinstitutional partnership, was based on key principles of collaborative development of a learning model to provide reciprocal benefits for our students. GHCR involves partnered medical student groups in our schools discussing real medical cases and relevant global health concepts in a virtual classroom using videoconferencing. Given international education partnerships have historically been unequitable, this study endeavoured to incorporate learning from existing literature on establishing and maintaining equitable collaborations [14, 23, 24]. Mutual respect and benefit, trust, good communication, and clear partner roles and expectations were key components in this collaboration [24, 25]. Lessons learned in the Aqoon study highlighted the importance of efficient communication channels and task sharing for mutual collaboration, and these were implemented into our intervention design [23]. This paper presents the mixed-method study on New Zealand and Samoan medical students’ self-reported learning and experiences in GHCR.

The two medical schools and countries involved in this study are from different cultural contexts. Samoa has a population of 197,097 with one tertiary level hospital, and a predominately rural population [26]. New Zealand has a population of 5,042,000 with eight tertiary and 19 secondary level hospitals, and a predominately urban population [27, 28]. Samoa ranks 105th in the United Nations Human Development Index whilst New Zealand ranks 9th [26, 29]. 23% of New Zealand’s government expenditure is on health, whilst Samoa spends 15% on health [29]. Physician density is nine-fold higher in New Zealand (3.061 per 1000) than in Samoa (0.344 per 1000) [26, 29]. Over the last few decades there has been major migration from Samoa to New Zealand as noted by the New Zealand Census, which shows 182,721 who culturally identify as Samoan in New Zealand [27].

## Methods

### Study design

We used a mixed methods research (MMR) design to triangulate sequentially collected quantitative and qualitative data. The mixed method approach provided greater breadth and depth of understanding regarding students’ self-reported learning and experiences in the GHCR [30, 31]. All participating students were invited to complete a post-GHCR questionnaire and students were randomly selected to participate in semi-structured interviews. The University of Otago Human Ethics Committee and National University of Samoa Research and Ethics Committee reviewed and approved this study.

### GHCR learning model

**Fig. 1:**
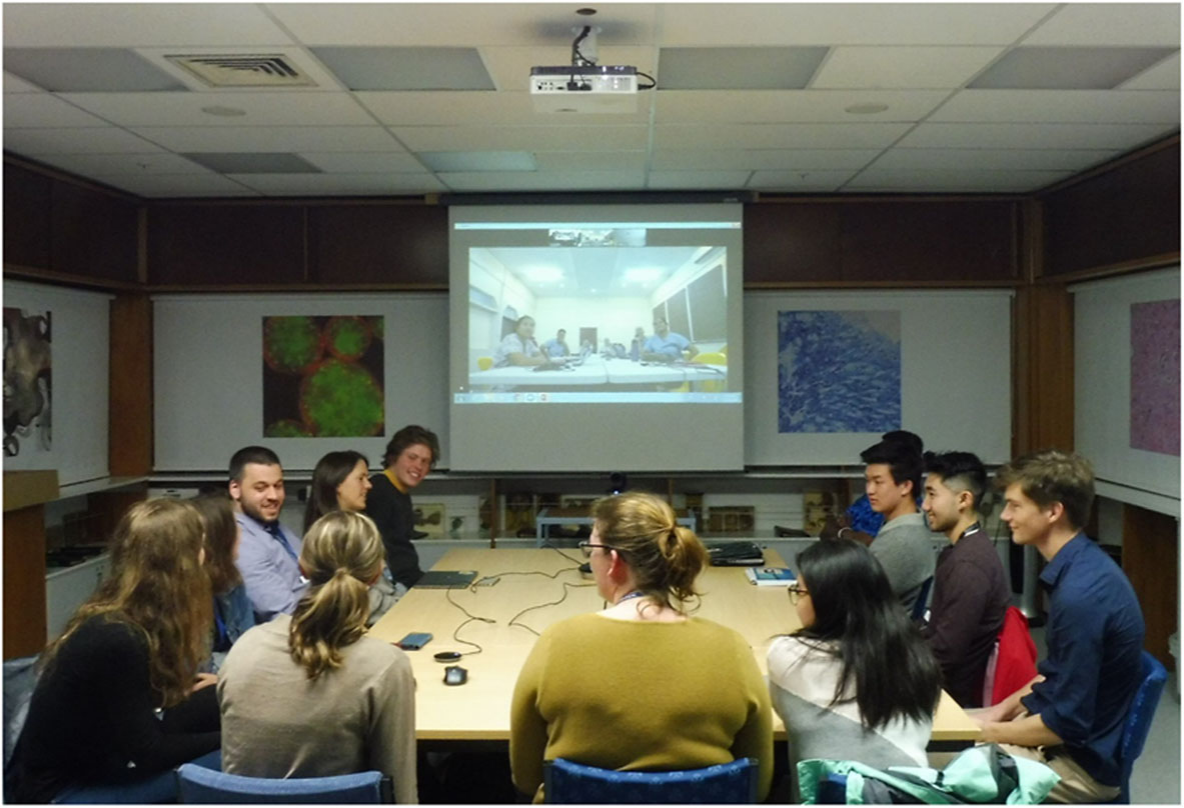
GHCR session underway showing New Zealand students with Samoan students on the screen

**Fig. 2:**
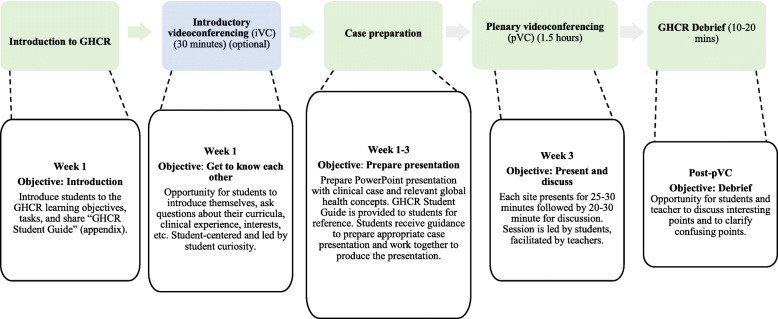
Learning design of the GHCR. iVC is optional. (iVC = introductory videoconferencing, pVC = plenary videoconferencing)

GHCR involves two small groups of medical students in different countries presenting and discussing medical cases and relevant global health concepts via a 90-min videoconference, followed by an off-line debrief at each centre (Figs. 1 and 2). Using a template, the “GHCR Student Guide”, each group prepares a standardised case presentation (Additional files 1 and 2), usually of a patient seen on a concurrent or recent clinical attachment. The template requires the case presentation to include associated global health concepts, such as the relevant determinants of health, cultural influences and practices affecting healthcare, as well as how the patient accessed the health system and was followed up. Global health concepts were sourced from existing literature on global health competencies published by Consortium of Universities for Global Health, Bellagio Global Health Education Initiative, and others [5, 7, 9]. Preparation of the case presentation is largely self-directed by the students, who distribute the global health topics amongst their group members.

The presentations typically include maps, diagrams and photographs so each group can introduce their international peers to the local hospital and healthcare systems. They also explain the availability and status of local transport, water, sewerage and other infrastructure for their patient. They compare the income, housing, and diet of the patient and family to their local community. Thus, students in each country are able to provide key local information and insights for global health learning related to the case presentations.

Zoom® has been used for video-conferencing due to its affordability, ease of use and the screen-sharing features. The videoconferences are facilitated by senior, final year medical students at each centre who have done GHCR previously. Teachers are present, but literally take the back seats once they have introduced themselves. The 90-min plenary videoconference (pVC) starts with students getting to know each other through a brief ice-breaker activity, coordinated by the senior students, who also clarify the objectives and structure of the pVC. Following this, the student groups from each side do their case-based presentations, typically, 25 min each. Then, 20–30 min of the videoconference is reserved for “unscripted” discussion between the two student groups. At the end of the videoconference there is a brief concluding plenary wrap-up discussion with input from teachers from both sides. The pVC is followed by a 10–15 min off-line local debriefing during which students are encouraged to reflect on the discussed topics and their experiences in GHCR, guided by the senior students and teachers.

An optional component of GHCR, which is not routinely used, is a 30-min introductory videoconference (iVC), a week or two prior to the pVC. It is intended for students to socialise with their international peers and discuss their experiences of being medical student colleagues in different health systems and countries.

### Settings and participants

This study collected and evaluated data from five GHCR sessions between NUS and OMS, occurring.

sequentially over one academic year from February to November 2017 (Additional file 3). All five GHCR sessions involved one NUS group (ten students) and one of five different OMS student groups, either a Christchurch campus group (usually 12–13 students) or a Dunedin campus group (usually 20 students). For the Samoan medical students at NUS, GHCR was integrated into the Community Health Module. GHCR is integrated into Year 4 Public Health Module for medical students at the Dunedin campus, and in the Year 5 Paediatrics Module at the Christchurch Campus. GHCR is a compulsory component of these curricula. Participation in this study and data collection was voluntary with written consent obtained from each participant prior to their GHCR.

### Data collection and analysis

The post-GHCR questionnaire consisted of a mix of multiple-choice, five-point Likert scale questions, and free-text responses (Additional file 4). Qualtrics® was used as the online survey platform. The questionnaires were electronically distributed by email to participants immediately after their GHCR with a two-week response period. Participants in the single NUS group received the post-GHCR questionnaire after their first GHCR. The face-to-face interviews were conducted by the lead investigator, RKB, using a semi-structured guide (Additional file 5). Interviewees were selected by non-probability sampling in all centres. Christchurch and Dunedin students were interviewed within 2 weeks of their GHCR, while Samoan students were interviewed after their third GHCR. Interviews were conducted until data saturation was reached in each centre. Audio recordings were transcribed verbatim and cross-verified for accuracy by the lead investigator and an administrator. Quotes are numbered according to participant interview (P) or questionnaire (Q) number.

The quantitative data were analysed using SPSS. Qualitative data were analysed using general inductive thematic analysis to identify pertinent patterns [32]. Quantitative and qualitative data analyses were conducted in two parts: the first was specific to each centre, and the second part analysed complementarity and divergence of data between centres. The learning outcomes reported by participants were categorised using the three levels of learning outlined by Frenk et al.: informative learning, formative learning, and transformative learning [3].

### Role of the funding source

The funders of the study had no role in the study design, data collection, data analysis, data interpretation, or writing of the report. RKB and APM had full access to all the data in the study and all authors had final responsibility for the decision to submit for publication.

## Results

The post-GHCR questionnaire response rate was 85% (74/87) (Table 1). Interviews were undertaken with six students from two Christchurch groups, seven students from two Dunedin groups, and six students from the single Samoa group.

**Table 1:** Response to the post-GHCR questionnaire by student group and location

Centre	Centre group	Group response to post-GHCR questionnaire	Overall centre response rate
UOC (39)	UOC-A (13)	13/13	82% (32/39)
	UOC-B (13)	10/13	
	UOC-C (13)	9/13	
DSM (38)	DSM-A (20)	20/20	84% (32/38)
	DSM-B (18)	12/18	
NUS (10)	NUS-A (10)	10/10	100% (10/10)

Students reported acquiring the intended learning outcomes relating to patient care, health systems, culture, and determinants of health with regards to their partner country. Qualitative data was indicative of attitudinal changes in relation to cultural humility and curiosity. The self-reported learning has been categorised into the learning levels outlined by Frenk et al.: informative learning, formative learning, and transformative learning (Table 2) [3]. Informative learning is about acquisition of knowledge and skills; formative learning involves socialising students around values in order to produce professionals; and transformative learning is about developing leadership attributes in order to produce enlightened change agents to address health inequities.

**Table 2:** Self-reported learning outcomes and experiences of medical students in the GHCR supported by quantitative and/or qualitative data. Qualitative data has been provided in Tables 3, 4 and 5

Learning outcomes		Quantitative	Qualitative
Informative	Patient Care	√	√
	Culture and impact on health	√	√
	Determinants of health	√	√
	Communication		√
	Research		√
Formative	Collaboration	√	√
	Curiosity	√	√
Transformative	Vision for progress		√
	Cultural humility		√
**Experiences**			
Innovation		√	√
Connectivity			√

Table 2 also shows two major themes regarding students’ experiences of participating in the GHCR, innovation and connectivity. All quantitative data are shown in Tables 3, 4 and 5. We show percent responses where data are derived from the questionnaire and illustrative quotes where data are derived from the interviews.

**Table 3:** Informative learning supported by quantitative data

Subtheme	Post-GHCR questionnaire statement	Likert-scale data (%)						Median
			Strongly agree	Agree	Neutral	Disagree	Strongly disagree	
Patient care	GHCR gave me insight into the differences in presentation and care of a common medical condition between Samoa and New Zealand.	Christchurch (*n* = 32)	19%	66%	12%	3%	0%	2
		Dunedin (*n* = 32)	26%	61%	13%	0%	0%	2
		Samoa (*n* = 10)	80%	20%	0%	0%	0	1
		Overall (*n* = 74)	30%	58%	11%	1%	0%	2
	GHCR Increased my understanding about global health measures to prevent and control a common medical condition in different healthcare settings.	Christchurch (*n* = 32)	19%	66%	12%	3%	0%	2
		Dunedin (*n* = 32)	26%	61%	13%	0%	0%	2
		Samoa (*n* = 10)	80%	20%	0%	0%	0%	1
		Overall (*n* = 74)	30%	58%	11%	1%	0%	2
Health systems and impact on health	Participating in the GHCR increased my understanding of the following aspects of global health, with regards to the other country: health system and impact on health outcomes.	Christchurch (*n* = 31)	16%	68%	6%	6%	3%	2
		Dunedin (*n* = 31)	16%	65%	16%	3%	0%	2
		Samoa (*n* = 10)	50%	50%	0%	0%	0%	1.5
		Overall (*n* = 72)	21%	65%	10%	4%	0%	2
Determinants of health	Participating in the GHCR increased my understanding of the following aspects of global health, with regards to the other country: socioeconomic and environmental impact on health.	Christchurch (*n* = 31)	23%	58%	16%	3%	0%	2
		Dunedin (*n* = 31)	6%	52%	19%	23%	0%	2
		Samoa (*n* = 10)	50%	50%	0%	0%	0%	1.5
		Overall (*n* = 72)	19%	54%	16%	11%	0%	2
	Participating in the GHCR increased my understanding of the following aspects of global health, with regards to the other country: barriers to accessing healthcare.	Christchurch (*n* = 31)	35%	48%	10%	6%	0%	2
		Dunedin (*n* = 31)	26%	65%	10%	0%	0%	2
		Samoa (*n* = 10)	50%	50%	0%	0%	0%	1
		Overall (*n* = 72)	33%	56%	8%	3%	0%	2
	The GHCR experience increased my understanding of the importance of knowing about the determinants of health.	Christchurch (*n* = 31)	6%	61%	19%	10%	3%	2
		Dunedin (*n* = 31)	16%	58%	6%	19%	0%	2
		Samoa (*n* = 10)	50%	50%	0%	0%	0%	1.5
		Overall (*n* = 72)	17%	58%	11%	13%	13%	2
Culture and impact on health	Participating in the GHCR increased my understanding of the following aspects of global health, with regards to the other country: cultural diversity and impact on health.	Christchurch (*n* = 31)	26%	65%	6%	3%	0%	2
		Dunedin (*n* = 31)	12%	55%	23%	10%	0%	2
		Samoa (*n* = 10)	60%	40%	0%	0%	0%	1
		Overall (*n* = 72)	25%	57%	12%	6%	0%	2
	The GHCR experience increased my understanding of the importance of knowing about how culture and health interact at a global level.	Christchurch (*n* = 32)	26%	52%	19%	0%	3%	2
		Dunedin (*n* = 32)	16%	61%	10%	13%	0%	2
		Samoa (*n* = 10)	60%	40%	0%	0%	0%	1
		Overall (*n* = 74)	26%	54%	13%	6%	1%	2

**Table 4:** Formative learning supported by quantitative data

Subtheme	Post-GHCR questionnaire statement	Likert-scale data (%)						Median
		Location	Strongly agree	Agree	Neutral	Disagree	Strongly disagree	
Collaboration	Collaborating with my international peers was valuable to my learning in the GHCR.	Christchurch (*n* = 32)	27%	50%	10%	10%	3%	2
		Dunedin (*n* = 32)	30%	47%	20%	3%	0%	2
		Samoa (*n* = 10)	60%	30%	10%	0%	0%	1
		Overall (*n* = 74)	33%	46%	14%	6%	1%	2
Curiosity	Participating in the GHCR has __________ my interest in learning about global health.		**Greatly increased**	**Increased**	**Neutral**	**Decreased**	**Greatly decreased**	
		Christchurch (*n* = 32)	9%	63%	25%	0%	3%	2
		Dunedin (*n* = 32)	6%	41%	47%	6%	0%	2
		Samoa (*n* = 10)	30%	60%	10%	0%	0%	2
		Overall (*n* = 74)	11%	53%	32%	3%	1%	2

**Table 5:** Innovation theme supported by quantitative data

Post-GHCR questionnaire statement	Data			
	Mode of learning	Mean	Mode	Median
How would you like to learn about global health? Please rank from 1 to 6 (1 being most desirable and 6 being least desirable)	GHCR	1.7	1.0	1.0
	In-house tutorial	2.8	2.0	3.0
	Collaborative case-based learning with medical students in your own country	3.1	2.0	3.0
	Lecture	3.9	4.0	4.0
	Personal reading (e.g. journal articles, books, etc.)	4.6	5.0	5.0
	E-learning (e.g. Coursera, etc)	4.8	6.0	5.0

### Reported learning outcomes in GHCR

#### Informative global health learning

Most students (88%) agreed that they gained insight into the differences in presentation and care of common medical conditions between Samoa and New Zealand (Table 3). This included learning about differences in investigations and treatments, and how availability of resources influenced overall care:

*I like comparing the treatments, the local treatments, and the NZ treatment, it's really good to have a look at what we are doing compared to that [New Zealand treatment].* (Samoa, P. 1)

*… the resources constraint is quite a big thing to keep in mind of things they would like to do but can’t because it more difficult for them. That just being able to order the test, that makes it a lot easier on us than for them. They have more diagnostic uncertainty which kind of came through in the case.* (Christchurch, P. 2)

*I knew that it would be tougher for the Samoan patients to get to hospital but didn't actually realise how much of a barrier it is for them.* (Christchurch, P. 6)

Overall, 86% of students agreed they learnt about their partner country’s health system and its impact on patient outcomes. Learning included aspects of initial community and primary-based care, differences in referral pathways for hospital care and follow-up:

*It is the care that New Zealand provides on discharge and the follow-up strategies, and the prevention is really good. Regarding when you compare it to us, here it's somewhat very simple.* (Samoa, P. 2)

*Having time for unscripted discussion really let us get an insight as to healthcare and promotion in Samoa, as well as what life is like for medical students.* (Dunedin, Q. 22)

Students commented that the clinical cases helped contextualise global health learning:

*Learning about the Samoan case helped contextualise the difference between our two health systems.* (Christchurch, Q. 3)

Most students (82%) agreed that GHCR increased their understanding of how culture influences health in their partner country (Table 3). Students reported how their increased understanding of culture provides insights into patients’ ideas, attitudes, and behaviours regarding healthcare:

*Personally, [the most valuable aspect of GHCR was] the impact culture has; whether good, bad, or none, on health.* (Samoa, Q. 10)

*I was really surprised that in Samoa traditional healers are so prominent. You see a patient you don’t understand why they are not taking their antibiotics … the reason behind that will be very different [compared to a New Zealand European].* (Christchurch, P. 3)

Several New Zealand students started with the assumption that the Samoan students would be similar to Samoans living in New Zealand, and were often quite surprised when they saw the difference, for example:

*And I don't know, I actually thought, this might make me sound like an idiot, but I actually thought “Ah, it will just be like people, you know, like the Samoans [that I know] in New Zealand” And, but it was quite different still, … they still have very strong cultural beliefs, and I think that came through … just even the way they did their presentation, the things they were prepared to talk about, you know because we were talking about sexually transmitted infections.* (Dunedin, P. 3)

Most (75%) students found that the GHCR increased their understanding of the importance of learning about the determinants of health to improve patient care (Table 3). Samoan students expressed changes in their perspective and greater understanding of the barriers to accessing healthcare for some Samoan patients:

*In the doctor’s head, it is always carelessness [by patients], but then you can’t understand it is the money, financial support, the transport. We have to look at all those factors, the factors that stop them from access to healthcare*. (Samoa, P. 5)

Of all students, 89% agreed that the GHCR increased their awareness of barriers to accessing healthcare:

*Things are really different just in terms of the amount of doctors they have who can service areas, like learning there were only three doctors for an entire population of people on one of their Islands was mind-blowing in a sense.* (Dunedin, P. 6)

Students also noted that across both countries there is commonality in the healthcare challenges and inequities:

*I guess also the striking similarities, even though New Zealand and Samoa seem a world apart, both struggle with access to healthcare, education around healthcare and inequity in health.* (Christchurch, Q. 1)

Students were required to collate patient information and relevant local health data for their structured case presentations.. Students reported that finding and explaining local data to their partner group helped them develop their research and analysis skills, as well as hone their presentation abilities:

*It made us re-evaluate how we do things because when you present to someone who understands the system you do it differently [compared to] when you present to someone who doesn’t.* (Christchurch, P. 2)

*Epidemiology is the most challenging aspect of the slides … because we have to find the raw data from the admissions books in the wards or the discharge summaries.* (Samoa, P. 6)

In summary, informative learning in the GHCR format enabled students to compare and contrast the clinical.

presentations and care of common medical conditions, the health systems, determinants of health and.

cultural impacts on healthcare between New Zealand and Samoa and discuss how these factors influence patient outcomes.

#### Formative learning related to global health in GHCR

Most students (79%) agreed that collaboration with their international peers in the GHCR was valuable to their learning. Comments from students in both countries indicated that the face-to-face, student-led format of the GHCR videoconference provided a collegial forum for socialising as health professionals-in-training. They expressed reciprocal commitment to each other’s learning and mutual respect:

*I like the idea that the classroom is about sharing our case with the Samoan students, and they would share their case with us.* (Dunedin, P. 4)

*Positive is learning from each other, learning about the different cultures.* (Samoa, P. 5)

This sense of collegiality enabled valuable opportunities for student discussions around cultural values and ethics, as well as equity:

*It was when they asked, “Why is this such a big difference between Māori (indigenous people of New Zealand) and European statistics?” and everyone looks at each other and how do you answer that question?* (Christchurch, P. 1)

*It was good to be prompted to think about how sexual health is a much more taboo subject in Samoa and how this impact on sexual health education and access to care.* (Dunedin, Q. 7)

Students particularly valued the spontaneous discussions that followed their formal presentations (Table 4). These peer interactions helped establish engagement between students in the virtual classroom:

*The classroom session as a whole is just great, but I think just getting to talk to people at the end outside of our scripted presentations was really cool, to have that back and forth, to talk about things as they came up, and that we found to be really interesting.* (Christchurch, P. 4)

Samoan and New Zealand students socialised with values of collegiality, equality and reciprocity, expressing mutual commitment to learn together:

*... a great learning experience for me. Understanding especially how culture, environment and health systems do affect healthcare immensely for different populations. The online video conferencing really does help in sharing similarities and differences.* (Samoa, Q. 10)

#### Transformative learning related to global health in GHCR

Students reported their interactions in GHCR exposed them to diverse opinions and new perspectives from.

their international peers. For some this triggered changes in their viewpoints and aspirations for their future.

medical practice. A number of Samoan students expressed a vision for progress to improve their own.

healthcare system and become agents of change:

*Primary health care is the change I want to improve here, going out to rural places, creating awareness programmes.* (Samoa, P. 1)

New Zealand and Samoan students recognised the importance of comparing their health systems in order to recognise the strengths of each country’s system, as well as areas for improvement for each:

*So, if we are able to spend more time listening to how things are done in other countries, I think that would increase our appreciation of what we have got here. And maybe change our mind about some of the things that we do for the better, and we can also help other people of course.* (Christchurch, P. 2)

*Perspective and insight on how to improve on healthcare services [were the most valuable aspects of GHCR]. Some things are done better in another country, which can be used to adapt new ideas for future development. (*Samoa, Q. 3*)*

For some students, GHCR provided an opportunity to view their own culture by gaining an outsider’s perspective:

*So, it was just one of those things, of like, becoming aware of your own culture through experiencing someone else’s culture.* (Dunedin, P. 1)

*I think every time that you are made aware of your own culture makes you realise how important culture identity and systems are.* (Dunedin, P. 3)

#### Student experiences in GHCR

Students found GHCR innovative and a “cool” and “tangible” way to learn about global health and broaden.

their perspectives. They found it more effective than didactic learning methods (Table 5):

*This is based on making global health seem like a more real and tangible thing (for lack of a better description). We talked to people who are in a different health system with different, but also surprisingly similar in some instances, health problems. It seems so much more accessible than learning about global health on a purely theoretical basis.* (Christchurch, Q. 1)

*I doubt it would have made as much of an impact if I had just read about it or had lecturer talk about it. I think student taught sessions is useful and can be more interesting and engaging.* (Dunedin, P. 1)

Students reported that the videoconference created a collegial, shared virtual classroom:

*Just the fact that we were talking and having a lesson with student in Samoa, and that is kind of special really, that we could break down geographical barriers with technology.* (Dunedin, P. 5)

*The fact that they were right there, and we could ask them questions live. It was really cool to see how our curriculums and lifestyles contrasted to theirs.* (Christchurch, Q. 26)

Samoan students particularly appreciated being able to learn about global health, without having to travel:

*It can be easily accessed through videoconferencing and we don’t have to travel with a lot of expense and a lot of other issues. But it’s something we can do from where we are, and we can be exposed. We can learn from each other over the videoconferencing.* (Samoa, P. 2)

Poor connectivity in audio and video streaming arose in several sessions. However, as long as the audio and shared desktop connections were maintained the sessions proceeded without significant problems and no sessions were abandoned due to inadequate connectivity.

## Discussion

The findings of this study demonstrate that GHCR is a promising model for internationally partnered medical schools to utilise for digitally enabled, student-led, case-based global health learning. The self-reported learning in GHCR aligns favourably with recommended global health learning competencies [7–9, 33]. Values considered important in global health are promoted in GHCR, with evidence of formative and transformative learning by some students. Students from both countries agreed GHCR provided mutual benefit, indicating reciprocity.

**Fig. 3:**
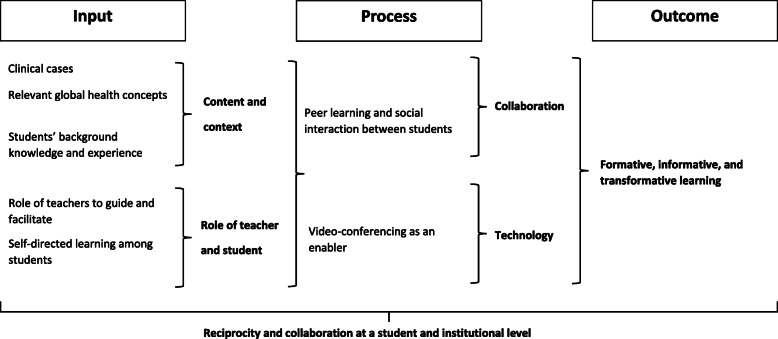
Key elements leading to the positive learning outcomes and experiences among students in the GHCR. Elements have been categorised into input and process

Figure 3 illustrates the elements that we consider key to positive learning outcomes and experiences in GHCR. The structured case-based presentations prepared by the students are the platforms for them to exchange their own local background knowledge, learning and experiences with their international peers during the videoconference. The case-based format is important because it provides windows to learn about global health concepts in a “real and tangible” manner, without having to travel. Insertion of global health learning topics into the case presentations, requires the students to personally introduce their country’s health system, cultural practices, and determinants of health to their international peers in the context of real cases.

The unscripted student-led discussions are particularly valued, enabling the students to reflect and interact as health professionals-in-training. Students critiqued and discussed a wide range of topics, including sensitive topics such as cultural taboos and persisting health inequities in their countries. Face-to-face interactions in the videoconference rapidly generate a strong sense of collegiality and commitment to collaborative learning. The role of teachers in GHCR was rarely commented on by students, which reflects our intention for a student-led format for the videoconference. Thus, teachers step back and take back seats, but are present during the videoconference should expert input be needed. As well, teachers are actively involved in the concluding off-line local debriefings which play an important role at the end of the session to promote student reflection, a key component of GHCR.

The self-reported global health learning outcomes of our students align with all three tiers of learning outlined by Frenk and colleagues. Regarding informative learning, students reported that they learned much from the structured case presentations because global health topics were linked to the case presentations.

This allows students to engage with global health topics in the context of the real situations and experiences of patients they have seen. Reciprocal case presentations allow comparisons of patient care, health systems, cultures, and determinants of health. The time for unscripted discussion between the students after the case-based presentations is highly regarded by students and valuable for formative learning, allowing them to socialise as doctors-in-training around professional and cultural values underlying healthcare and practice. Students reported that exposure to their international peers’ different perspectives and opinions prompted them to reflect on and become more aware of their own health system and culture. For some students, GHCR promoted transformative learning with evidence of cultural curiosity and humility. Some indicated that GHCR gave them a vision for progress for their own health system and aspirations to be agents of change. Thus, the GHCR format capitalises on the capability of digital technologies to facilitate peer learning through face-to-face interactions of the students with their international peers in a shared virtual classroom.

GHCR achieves many of the benefits predicted by Frenk and colleagues in their recommendations for the adoption of digital technologies in health professional education, especially in global health, to transcend international boundaries and facilitate inter-institutional collaborations for mutual advance [3]. The GHCR collaboration between our partnered institutions has provided reciprocal and equitable benefits for our students; this is a key goal for global health learning within international health education partnerships [3, 14]. It was the result of close interactions and collaborations between teachers at the Otago Medical School and National University of Samoa in the development and implementation of GHCR, all relying on digital technologies.

Our study findings are consistent with other published digitally enabled global health learning models such as RIPPLE and Aqoon, which also found that students appreciated learning alongside their international peers. However, these models were either pilot studies and, to our knowledge, have not been integrated into the curriculum for all students [21–23]. GHCR has been integrated into our respective curricula since 2017. Linking global health learning to concurrent clinical case studies, as occurs in the GHCR model, enables integration of global health learning into multiple points in medical curricula, without necessitating significant new curricular time or resource demands.

One of the strengths of this study is the mixed methods approach, which yielded rich data enabling us to explore the breadth and depth of student learning and experiences. These were elaborated on and clarified by students in the semi-structured face-to-face interviews in all three locations. Previous studies on learning models similar to GHCR have employed primarily quantitative research methods. Although easier to conduct, such methods do not allow such in-depth exploration of the perceptions of the students regarding their experiences and the effectiveness of the model for their global health learning [21, 22].

This study has several limitations. Although uncommon and rarely disruptive in this study, poor connectivity in audio and video streaming is a limitation of this learning model. A contingency plan was devised to ensure the collaboration continued, for example when video streaming was problematic, we reverted to audio only. Also, this study relied on self-reported data, which may have led to over- or under emphasis of learning outcomes and experiences. Although the findings were consistent across each of the GHCR sessions and three centres, the relatively small sample size and disproportionate number of New Zealand students to Samoan students may be a limitation. As well, the same Samoan student group collaborated with several different New Zealand groups. As the Samoan students became more accustomed to the GHCR format, their increased ease and confidence in the sessions may have had a positive influence on the experiences and learning of the New Zealand medical students. In future research of the global health classroom, it will be helpful to collect descriptive data about participants, such as age, gender, and past knowledge or experience with global health, to better understand and contextualize the findings.

The Samoan students were consistently more in agreement in response to the survey questions than the New Zealand students (Tables 3, 4 and 5). Although there may be cultural elements on both sides underlying these differences, the Samoan students did also comment on additional benefits to them from GHCR. Because of limited health data available to the Samoan students, GHCR required them to develop health data collection and analysis skills for their presentations. As well, Samoan students appreciated the relative freedom to express themselves in the GHCR peer discussions. Several commented positively on increased confidence and capability in their communication skills following multiple GHCR sessions.

Since our study was undertaken, medical education has undergone major disruptions and adaptations because of the COVID-19 pandemic [34, 35]. Medical education has needed to become more flexible, virtual, and adaptable [34, 36]. Our model presents an opportunity for medical education to evolve under these new circumstances. Developing global health partnerships using technology may leave a lasting positive impact for the future of medical education.

## Conclusion

In view of the positive findings and outcomes from our study of GHCR we present it as a readily adaptable digitally-enabled model for equitable, collaborative global health learning between students in internationally partnered institutions. Students acquired intended learning outcomes relating to patient care, health systems, culture, and determinants of health with regards to their partner country. GHCR also consolidated student learning about these topics with regard to their own country. It provided a collegial forum for formative learning and, for some students, for transformative learning. Students from both countries reported mutual benefit indicating reciprocity. Future research could explore learning outcomes and experiences of medical students from other countries and cultures, as well critique this collaboration using established metrics for equitable partnerships. This may help to further develop and strengthen the adaptability and feasibility of this learning model. Further comparison of GHCR with similar models, such as Aqoon and RIPPLE, may help elucidate the key elements to improve the model. In a subsequent article, we aim to present the lessons learned in developing and implementing the Global Health Classroom at our medical schools in New Zealand, Fiji, Samoa, and Mexico. That paper will elaborate on how we formed our partnership based on reciprocity and collaboration and will be relevant to educators and clinicians wishing to expand their own global health practice and curricula.

## Supplementary Information

**Additional file 1.** Global Health Classroom Student Guide.

**Additional file 2.** Summary of the Student Presentation Template.

**Additional file 3.** Summary of the GHCR Case Presentation Topics.

**Additional file 4.** Post GHCR Questionnaire.

**Additional file 5.** Interview Guide for GHCR Study.

## Data Availability

All data supporting our findings is contained in this article and its supplementary information files.
