# Supplementary material for: Global health classroom: mixed methods evaluation of an interinstitutional model for reciprocal global health learning among Samoan and New Zealand medical students
Source: Global Health. 2021 Sep 3;17:99. doi: 10.1186/s12992-021-00755-8 (PMC8414472; doi:10.1186/s12992-021-00755-8)
Supplement: Supplementary file 1 — Additional file 1. Global Health Classroom Student Guide. [file 12992_2021_755_MOESM1_ESM.docx]

Additional file 1. Global Health Classroom Student Guide.

As a group please prepare the PowerPoint Presentation with the following information. One pair of students will select a real clinical case to present on. Everyone else choose one of the guiding questions below and follow the instruction in brackets strictly. Each guiding question should be 1 slide, presented for 1-2 minutes.

Please use the bullet points under each key heading to guide your research and presentation – you may present on other points if they are more interesting and relevant to your clinical case!

1. Patient information (4-5 slides, present for 10-15 mins
   - Presenting complaint, History of presenting complaint, past medical history, social history, family history, examination findings, differential diagnosis, investigation, diagnosis and treatment, outcome, and prognosis.
2. Epidemiology of your case (1 slide, present for 1-2 mins, 2 students)
   - National prevalence/incidence data; age gender and ethnic prevalence/incidence; risk factor prevalence/incidence; usual causes (common organism in your country); compare to global data.
3. Preventive and awareness measures related to your case (1 slide, present for 1-2 mins, 2 students)
   - Primary prevention vaccinations (immunization schedule in your country); public health contact screening; risk factor prevention; awareness campaigns (e.g. Posters, TV ads, etc.), antibiotic resistance.
4. Accessibility and affordability of healthcare (1 slide, present for 1-2 mins, 2 students)
   - If somebody at home gets sick what do you do?
   - Delayed treatment – how and why; inadequate treatment – why and how
   - Determinants to accessing care: affordability, accessibility, health literacy, etc.
5. Cultural awareness (1 slide, present for 1-2 mins, 2 students)
   - Ethnic group diversity, languages, cultural/belief systems; sensitive topics; patient’s attitudes to doctors, health seeking behaviour, use of alternative medicine and treatment, lifestyle, and substance misuse.
