# Supplementary material for: Global health classroom: mixed methods evaluation of an interinstitutional model for reciprocal global health learning among Samoan and New Zealand medical students
Source: Global Health. 2021 Sep 3;17:99. doi: 10.1186/s12992-021-00755-8 (PMC8414472; doi:10.1186/s12992-021-00755-8)
Supplement: Supplementary file 2 — Additional file 2. Summary of the Student Presentation Template. [file 12992_2021_755_MOESM2_ESM.docx]

Additional file 2. Summary of the Student Presentation Template

| **Question** | **Student/s** | **Time and number of slides allocated** |
| --- | --- | --- |
| Patient presentation | 1-2 students | 15-20 minutes, 4-6 slides |
| Epidemiology | 1-2 students | 1-2 minutes, 1 slide |
| Referral system | 1-2 students | 1-2 minutes, 1 slide |
| Preventive and awareness measures | 1-2 students | 1-2 minutes, 1 slide |
| Accessibility and affordability | 1-2 students | 1-2 minutes, 1 slide |
| Cultural awareness | 1-2 students | 1-2 minutes, 1 slide |
