# Supplementary material for: Global health classroom: mixed methods evaluation of an interinstitutional model for reciprocal global health learning among Samoan and New Zealand medical students
Source: Global Health. 2021 Sep 3;17:99. doi: 10.1186/s12992-021-00755-8 (PMC8414472; doi:10.1186/s12992-021-00755-8)
Supplement: Supplementary file 3 — Additional file 3. Summary of the GHCR Case Presentation Topics. [file 12992_2021_755_MOESM3_ESM.docx]

Additional file 3. Summary of the GHCR Case Presentation Topics

| **GHCR** | **Clinical case** |  |
| --- | --- | --- |
| Christchurch-A  Samoa-A | Failure to thrive  Malnutrition |  |
| Dunedin-A  Samoa--A | Pelvic inflammatory disease |  |
| Christchurch -B  Samoa-A | Enuresis  Neonatal sepsis |  |
| Dunedin-B  Samoa-A | Pancreatitis |  |
| Christchurch -C  Samoa- A | Child with painful hip  Neonatal jaundice |  |
