# Supplementary material for: Global health classroom: mixed methods evaluation of an interinstitutional model for reciprocal global health learning among Samoan and New Zealand medical students
Source: Global Health. 2021 Sep 3;17:99. doi: 10.1186/s12992-021-00755-8 (PMC8414472; doi:10.1186/s12992-021-00755-8)
Supplement: Supplementary file 4 — Additional file 4. Post GHCR Questionnaire. [file 12992_2021_755_MOESM4_ESM.docx]

Additional file 4 Post GHCR Questionnaire

1. Where are you located?

- Samoa (1)
- Christchurch, New Zealand (2)
- Dunedin, New Zealand (3)

2. What is your current year of medical study?

- 4th (1)
- 5th (2)
- 6th (3)

***The following questions are based on your global health learning in the Global Health Classroom (GHCR).***

3. How interested were you in learning about global health prior to the GHCR?

- Very interested (1)
- Interested (2)
- Neutral (3)
- Uninterested (4)
- Very uninterested (5)

4. Participating in the GHCR has __________ my interest in learning about global health.

- greatly increased (1)
- increased (2)
- not changed (3)
- decreased (4)
- greatly decreased (5)

5. Please comment on your answer to the above questions. This question is optional. You may skip this question.

6. The GHCR experience enhanced my understanding of global health issues.

- Strongly agree (1)
- Agree (2)
- Neither agree nor disagree (3)
- Disagree (4)
- Strongly disagree (5)

7. GHCR gave me insight into the differences between presentation and care of a common medical condition in Samoa and New Zealand.

- Strongly agree (1)
- Agree (2)
- Neither agree nor disagree (3)
- Disagree (4)
- Strongly disagree (5)

8. GHCR increased my understanding about global health measures to prevent and control a common medical condition in different healthcare settings.

- Strongly agree (1)
- Agree (2)
- Neither agree nor disagree (3)
- Disagree (4)
- Strongly disagree (5)

9. Please comment on your answer to the above questions. This question is optional. You may skip this question.

10. Please rate the statement "Participating in the GHCR increased my understanding of the following aspects of global health, with regards to the other country."

|  | Strongly agree (1) | Agree (2) | Neither agree nor disagree (3) | Disagree (4) | Strongly disagree (5) |
| --- | --- | --- | --- | --- | --- |
| a) Socioeconomic and environmental impact on health (1) |  |  |  |  |  |
| b) Health system and impact on health outcomes (2) |  |  |  |  |  |
| c) Cultural diversity and impact on health (3) |  |  |  |  |  |
| d) Barriers to accessing healthcare (4) |  |  |  |  |  |

11. Please rate the statement "The GHCR experience increased my understanding of the importance of knowing about _________."

|  | Strongly agree (1) | Agree (2) | Neither agree nor disagree (3) | Disagree (4) | Strongly disagree (5) |
| --- | --- | --- | --- | --- | --- |
| a) how environment and health interact at a global level (1) |  |  |  |  |  |
| b) the determinants of health (2) |  |  |  |  |  |
| c) how culture and health interact at a global level (3) |  |  |  |  |  |

12. Please comment on your overall learning in the GHCR. What aspect of the GHCR helped you learn that?

13. Please comment on the most valuable aspects of the GHCR.

14. What aspects of the GHCR would you most want to change?

15. Please comment on your answer to the above questions. This question is optional. You may skip this question.

***The following questions are based on your experience of the GHCR learning design.***

16. The aims and process of the GHCR were clearly explained.

- Strongly agree (1)
- Agree (2)
- Neither agree nor disagree (3)
- Disagree (4)
- Strongly disagree (5)

17. How much time did you spend on the following tasks in this GHCR experience?

|  | < 30 mins (1) | 30 - 60 minutes (2) | 60 - 120 minutes (3) |
| --- | --- | --- | --- |
| Case preparation and research of guiding questions (1) |  |  |  |

18. The introductory and task briefing videoconferencing was a good way to start the GHCR.

- Strongly agree (1)
- Agree (2)
- Neither agree nor disagree (3)
- Disagree (4)
- Strongly disagree (5)

19. Would you prefer the 30-minute introductory and task-briefing GHCR session with or without videoconferencing?

- I would prefer the introductory and task briefing GHCR session without videoconferencing. (1)
- I would prefer the introductory and task briefing GHCR session with videoconferencing. (2)

20. Please comment on your answer to the above questions. This question is optional. You may skip this question.

21. Collaborating with my classmates was valuable to my learning in the GHCR.

- Strongly agree (1)
- Agree (2)
- Neither agree nor disagree (3)
- Disagree (4)
- Strongly disagree (5)

22. Collaborating with my international peers was valuable to my learning in the GHCR.

- Strongly agree (1)
- Agree (2)
- Neither agree nor disagree (3)
- Disagree (4)
- Strongly disagree (5)

23. I would have found it more valuable to have had a lecture on global health instead of the GHCR for my global health learning.

- Strongly agree (1)
- Agree (2)
- Neither agree nor disagree (3)
- Disagree (4)
- Strongly disagree (5)

24. Would you have liked a formal lecture about the healthcare system of the other country prior or during the GHCR case studies?

- Yes (1)
- Do not know (2)
- No (3)

25. Would you have liked a formal lecture about the culture of the other country prior or during the GHCR case studies?

- Yes (1)
- Do not know (2)
- No (3)

26. How would you like to learn about global health? Please rank from 1 - 6 (1 being most desirable way to learn and 6 being most undesirable way to learn global health).

______ Lecture (1)

______ In-house tutorial (2)

______ Global Health Classroom: collaborative case-based learning with medical students in another country (3)

______ collaborative case-based learning with medical students in your own country (4)

______ Personal reading (e.g. journal articles, books, etc.) (5)

______ E-learning (e.g. Coursera, etc.) (6)27. Please comment on your answer to the above questions. This question is optional. You may skip this question.

28. Any other comments/thoughts regarding GHCR that was not covered in this questionnaire?  This question is optional. You may skip this question.
