# Supplementary material for: Global health classroom: mixed methods evaluation of an interinstitutional model for reciprocal global health learning among Samoan and New Zealand medical students
Source: Global Health. 2021 Sep 3;17:99. doi: 10.1186/s12992-021-00755-8 (PMC8414472; doi:10.1186/s12992-021-00755-8)
Supplement: Supplementary file 5 — Additional file 5. Interview Guide for GHCR Study. [file 12992_2021_755_MOESM5_ESM.docx]

Additional file 5 Interview Guide for GHCR Study

1. *Can you tell me about your interest in global health?*

Prompts

*What are you interested in?*

*Why are you interested?*

*Do you think global health should be taught in our curriculum? Why and How?*

1. *Can you describe your experience of the global health classroom with Samoa/Nepal?*

Prompts

*What did you learn from it? Explore this.*

*Which aspects of the GHCR helped you learn what you have told me about?*

1. *Tell me your views on learning with students from other countries*

Prompts

*What were the positive and negative aspects? Why?*

1. *Tell me your views on learning in a cross-cultural virtual setting like the Global Health Classroom.*

Prompt

*What were the positive and negative aspects? Why?*

1. *What impact has the GHCR experience had on your perspectives?*

Prompt

*What aspects had what impacts?*

1. *Based on what we have talked about was your overall experience in the GHCR positive or negative?*

Prompts

*Why was it positive/negative?*

*Do you have any ideas or suggestions to make future global health classrooms better?*

We are near the end of our interview.

*Do you have any ending comments to make? Have I missed anything you consider to be important?*

Thank you very much for you time and sharing your perspective.
